# Modification of Premises for the Black Hole Information Paradox Caused by Topological Constraints in the Event Horizon Vicinity

**DOI:** 10.3390/e26121035

**Published:** 2024-11-29

**Authors:** Janusz Edward Jacak

**Affiliations:** Institute of Theoretical Physics, Wrocław University of Science and Technology, Wyb. Wyspiańskiego 27, 50-370 Wrocław, Poland; janusz.jacak@pwr.edu.pl

**Keywords:** black hole physics, non-thermal radiation mechanisms, information paradox

## Abstract

We demonstrate that at the rim of the photon sphere of a black hole, the quantum statistics transition takes place in any multi-particle system of indistinguishable particles, which passes through this rim to the inside. The related local departure from Pauli exclusion principle restriction causes a decay of the internal structure of collective fermionic systems, including the collapse of Fermi spheres in compressed matter. The Fermi sphere decay is associated with the emission of electromagnetic radiation, taking away the energy and entropy of the falling matter without unitarity violation. The spectrum and timing of the related e-m radiation agree with some observed short giant gamma-ray bursts and X-ray components of the luminosity of quasars and of short transients powered by black holes. The release of energy and entropy when passing the photon sphere rim of a black hole significantly modifies the premises of the information paradox at the falling of matter into a black hole.

## 1. Introduction

Since the formulation of black hole termodynamics initiated by the illuminating papers by Hawking and Bekenstein [1,2] about the entropy of a black hole, the problem of the fate of the information (related to entropy) encoded in matter falling into a black hole is still at the center of interest [3]. The proposed Hawking–Unruh radiation [1,4] has been proven to be fully random [5], and thus could not carry out the information specific to the matter consumed by the black hole, which causes an information paradox related to the irreversible loss of this information. The proposed next concept of the creation near the event horizon of particle–antiparticle pairs, with one carrying positive energy to infinity and the other carrying negative energy into the black hole (leading to its evaporation), does not solve the problem because of the quantum entanglement property called monogamy (particles cannot simultaneously participate in double entanglement). Particles and antiparticles are entangled, and Hawking radiation must be also entangled in consecutive time instants (a new radiation with an old one) [6]; hence, to avoid such multiple entanglement, a breaking of the particle–antiparticle entanglement at the event horizon of a black hole has been suggested. Immediately, broken entanglement between the infalling and outgoing partner particles would release energy, creating in this way a searing firewall at the event horizon of a black hole [6]. Neither Hawking radiation nor firewall have been observed as of yet. Some observational evidence of fuzzy black hole horizon searched in gravitational wave signature obtained from LIGO observations of bouncing black holes [7] were finally excluded because they occurred beneath the sufficient statistical significance level [8].

The information paradox remains at the center of the debate on quantum gravity, and various attempts are being made to reconcile it with quantum unitarity. There are searching effects which would be able to explain a unitary component of the thermal Hawking radiation [9,10,11,12,13,14]. In [9], the particle–antiparticle mechanism is repeated with time symmetry broken but with the negligence of entanglement monogamy. In [10,11] several assumptions are made about black hole quantization toward relativistic quantum mechanics and string theory [11], which might not be evident from the point of view of proper (yet not formulated) quantum gravity. More explicit quantization of black holes in terms of the gravitational Bohr-like atom has assumed a potential for the black hole Schrödinger equation as ∼$-\frac{1}{r}$ (where *r* is the distance to the gravitational singularity) [14], which is, however, with the negligence of the spacetime curvature and related term ∼$-\frac{1}{r^{3}}$ dominating the potential near the event horizon. In the latter potential, no bounds from below stationary states (atomic-like) exist (they do not exist for any exponent α>2 in potential $-\frac{1}{r^{\alpha }}$, for which a particle unconditionally falls onto the center, not creating an atom-like structure). Thus, arguing about quantum hairs or the atom-like model of black holes is not complete in the view of general relativistic physics. Nonetheless, the idea of quantum hairs due to gravity is popular; it needs, however, a further development of gravity quantization. In addition, from observations of gravitational waves [7], no signature of quantum hairs of black holes has been noticed [8].

In the present paper, we will contribute to the current discussion on the information paradox for black holes [1,3,6], taking advantage of the observation that close to the event horizon of a black hole, the homotopy class of particle trajectories changes itself qualitatively, and this topological property causes a local perturbation of quantum statistics in any multi-particle collective system of indistinguishable identical particles passing inward from the rim of the photon sphere (in the distance of $1.5\;r_{s}$ from a central singularity of a Schwarzschild black hole, where $r_{s}$ is the event horizon radius) [15]. The related quantum collective statistics transition is accompanied by the release of energy and information due to the local departure from Pauli exclusion principle constraints imposed on matter when passing the photon sphere rim. In particular, the Fermi spheres of fermions in dense quantumly degenerated multi-particle systems of identical indistinguishable fermions collapse, which is associated with the emission of electromagnetic radiation.

Below, we present this effect in more detail, including the simulation of the Fermi sphere decay at the collapse of 2.3 $M_{⊙}$ neutron star merger (at Tolman–Oppenheimer–Volkoff (TOV) stability limit [16,17]) associated with the release of ca. ∼$10^{47}$ J of electromagnetic radiation in a sub-second time period in agreement with some observed short giant gamma-ray bursts. Such bursts are equivalent to the conversion of a mass into the electromagnetic radiation energy with the efficiency of ca. 30%, highly exceeding the efficiency of nuclear fusion in stars, being of the order of 0.7%. A similar extreme efficiency of the mass-to-radiation conversion, reaching 30%, can be attributed also to the collapse of Fermi spheres of electrons and protons in accretion discs passing the photon sphere rim of superluminous quasars powered by giant black holes ∼$10^{9}$ $M_{⊙}$ and consuming ca. 10 $M_{⊙}$ per year [18]. The decay of Fermi spheres of electrons and protons in a stable flow of the highly compressed plasma in an accretion disc passing the photon sphere rim supplements the total luminosity of a quasar accretion disc with ∼$10^{40}$ W [18,19,20,21] of the non-thermal radiation from the vicinity of the event horizon not previously accounted for in conventional models of matter accretion by black holes. This non-thermal radiation is concentrated mostly in the spectral range of γ radiation (up to GeV) without a need for the Comptonization (inverse Compton effect in hot plasma) of soft thermal photons in more remote regions of an accretion disk conventionally assumed to fit astrophysical observations [19,22]. This allows to avoid the problem of the improbable and extremely high temperature of plasma needed for photon Comptonization in the vast accretion discs of giant quasars.

We point out the significance of the described quantum statistics transition when passing the photon sphere rim for the problem of an information and entropy behavior (information paradox [1,2,4,5] and firewall concept [3,6]) for matter falling into black holes.

The paper is organized as follows. In the following paragraphs of Methods (Section 2), we detail the topological effect in the vicinity of general relativistic gravitational singularity. Next, within Results (Section 3), using quantum mechanics approach, we draw out a conclusion related to the transition in quantum statistics in multi-particle systems passing the photon sphere rim and rooted in the trajectory homotopy qualitative local change in this region. The Section 4 embraces a consideration of the high-energy effects associated with the described quantum statistics transition and their comparison to astrophysical observations. Section 5 for the premises of the information paradox and the concept of a black hole firewall are finally presented. Some quantum mechanics methods needed for the reasoning in the developed model are summarized in the Appendices, including topology-type foundations for quantum statistics in collective systems of indistinguishable particles subjected to external constraints.

## 2. Methods

### 2.1. Change in Trajectory Homotopy Class When Passing the Photon Sphere Rim

Quantum topological effects are rooted in the algebraic topology [23] properties of the classical counterparts of quantum systems and expressed by homotopy groups related to the manifolds on which the dynamics of the particular classical system takes place [24]. When quantum effects concern the quantum statistics of identical, indistinguishable particles, then the homotopy group $\pi_{1}$ (frequently called the fundamental group) of the multi-particle classical configuration space for the specific manifold on which the collective multi-particle system resides is of primary importance [25]. The $\pi_{1}$ group of the multi-particle configuration space is called the braid group [25,26,27,28,29] and collects non-homotopic classes of closed loops of multi-particle trajectories in the configuration space, including the indistinguishability of identical particles (non-homotopy means that loops from different classes cannot be continuously deformed one to another). The braid groups allow us to distinguish between various quantum partners of the same classical particles like of fermions, bosons, anyons and composite topological particles (e.g., composite fermions or composite bosons). The difference between these types of quantum particles is distinct and specific for each type of scalar unitary representation of the classical braid group, which describes all exchanges of indistinguishable particles on some manifold (the domain of classical particle dynamics) because the $\pi_{1}\left(A\right)$ group is the collection of closed loops in the space A. If *A* is the multi-particle configuration space of *N* indistinguishable particles (as for braid groups), then bundles of *N* individual particle trajectories linking particle distributions, which differ only in the various numbering of particles, are closed loops due to the indistinguishability of particles—thus, braids display all possible exchanges of particles. Quantum multi-particle wave functions must transform according to the scalar unitary representation of a particular braid (an element of the braid group), the one which defines some pattern of particle exchanges (the renumbering of them). Though the renumbering of the arguments of the multi-particle wave function is a simple permutation of argument indices, the corresponding braid is not this permutation, in the general case, and includes also topological constraints specific to the manifold on which the particles are located. This reveals the notion of quantum statistics associated with the unitary representations of braid groups rather than of permutation groups (but for 3D manifolds braid groups equal to permutation groups). Braid groups have usually many different scalar unitary representations; thus, to the same classical particles, different quantum ones may correspond. For 3D manifolds on which classical particles can be located, the braid groups are always permutation groups [25], with only two possible different scalar unitary representations, symmetric and antisymmetric, describing bosons and fermions, respectively. For 2D manifolds, the braid group is different—called Artin groups (manifold being the 2D plane [30]) with more numerous scalar unitary representations giving rise to anyons, particles with fractional statistics [29,31,32]. More details are given in Appendix A including also the precise definition of quantum statistics in terms of Feynman path quantization [27,33,34].

The general relativistic curvature of the spacetime gives also an occasion to study the quantum statistics of indistinguishable identical particles on manifolds with various locations with respect to the gravitational singularity of a classical black hole. Changes in the homotopy properties of multi-particle trajectories in local manifolds approaching the event horizon of a black hole translate into quantum phenomena experienced by any matter built of indistinguishable particles and being consumed by black holes. To gain insight into such effects, the homotopy of classical trajectories of particles approaching the black hole event horizon must be first analyzed.

Let us consider the availability of local closed loops for trajectories of particles in various regions of the upper vicinity of the event horizon for a local multi-particle system of free massive identical and indistinguishable particles suitable to define their quantum statistics. The analysis of the trajectory behavior of free particles resolves itself by the consideration of geodesics in the Schwarzschild metric [35],

(1) $$\begin{array}{c} -c^{2}d\tau^{2}=-\left(1-\frac{r_{s}}{r}\right)c^{2}dt^{2}\\ +{\left(1-\frac{r_{s}}{r}\right)}^{-1}dr^{2}+r^{2}\left(d\theta^{2}+sin^{2}\theta d\phi^{2}\right), \end{array}$$

with $r_{s}=\frac{2GM}{c^{2}}$ is the Schwarzschild radius (event horizon radius) for the black hole with the mass *M* (*G*—the gravitational constant, *c*—light velocity in vacuum). Geodesics can be found via the conventional solution (cf. Ref. [36]) of the Hamilton–Jacobi equation for a particle with the small mass *m* in spherical rigid stationary coordinates (r,θ,ϕ, the same as for a remote observer, in which the Schwarzschild metric (1) is written; τ in this equation is the proper time of the particle). These trajectories (considered in the θ=0 plane without any loss of generality because of the rotational symmetry) have the form [36], for the trajectory radius,

(2) $$\begin{array}{c} ct=\frac{E_{0}}{mc^{2}}\times \int \frac{dr}{\left(1-\frac{r_{s}}{r}\right)\sqrt{{\left(\frac{E_{0}}{mc^{2}}\right)}^{2}-\left(1+\frac{L^{2}}{m^{2}c^{2}r^{2}}\right)\left(1-\frac{r_{s}}{r}\right)}}, \end{array}$$

and for the azimuthal angle,

(3) $$\phi =\int dr\frac{L}{r^{2}}{\left[\frac{E_{0}^{2}}{c^{2}}-\left(m^{2}c^{2}+\frac{L^{2}}{r^{2}}\right)\left(1-\frac{r_{s}}{r}\right)\right]}^{-1/2},$$

where L and $E_{0}$ are particle angular momentum and energy (integrals of the motion), respectively. Equation (2) is often rewritten in a differential form,

(4) $$\frac{1}{1-r_{s}/r}\frac{dr}{cdt}=\frac{1}{E_{0}}{\left[E_{0}^{2}-U^{2}\left(r\right)\right]}^{1/2},$$

with the effective potential,

(5) $$U\left(r\right)=mc^{2}{\left[\left(1-\frac{r_{s}}{r}\right)\left(1+\frac{L^{2}}{m^{2}c^{2}r^{2}}\right)\right]}^{1/2}.$$

**Figure 1:**
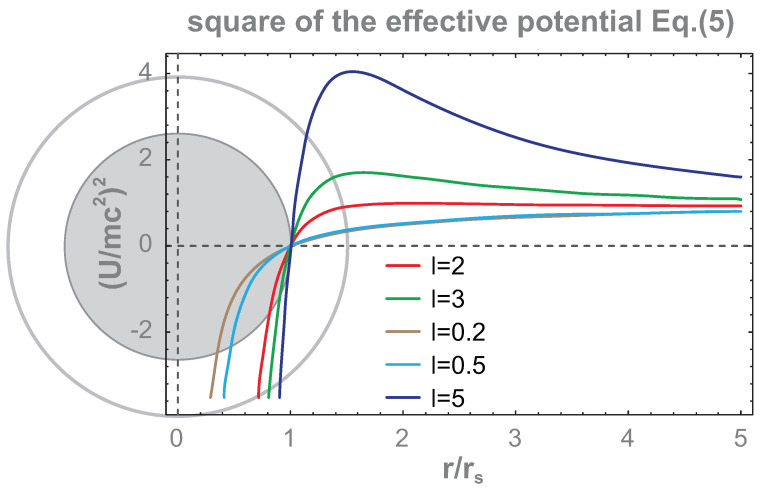
The squared effective potential (5) for some exemplary values of reduced angular momentum $l=\frac{L}{mcr_{s}}$ (dimensionless). With the increase in L, the squared potential is steeper in the region $r\in (0,1.5r_{s})$ (singular at r=0). The event horizon at $r=r_{s}$ and the innermost unstable circular orbit with $r=1.5r_{s}$ are marked.

The square of this potential is plotted in Figure 1, illustrating the main features of the black hole singularity neighborhood. From this figure, we note that the squared potential is steeper at $r<1.5\;r_{s}$ the greater the angular momentum L (this property of the folded spacetime near a black hole is often interpreted such that close to the event horizon, the centrifugal force additionally attracts a particle, quite oppositely to the classical Newton gravitation center).

**Figure 2:**
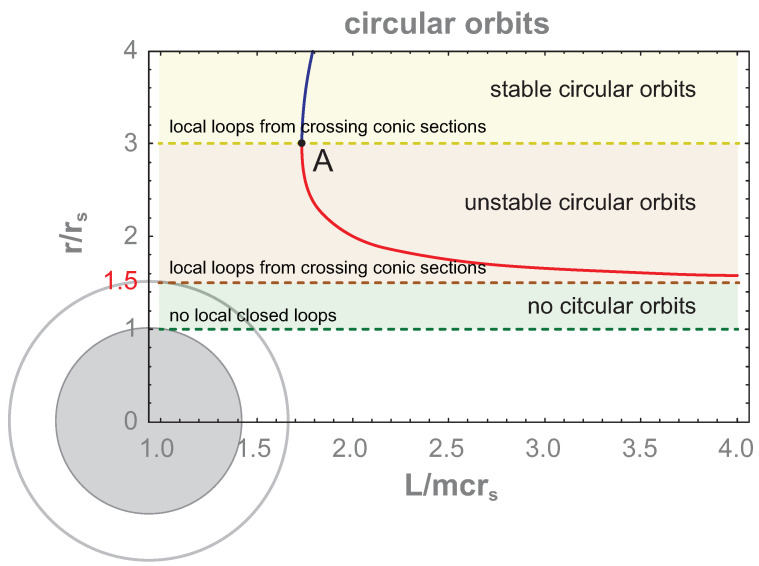
Radii of stable (blue line) and unstable (red line) circular orbits versus an angular momentum of a particle near the general relativistic gravitational singularity in the Schwarzschild metric (1) obtained by the solution of the second equation in the pair (6). The innermost stable circular orbit occurs at $r=3r_{s}$ (yellow dashed horizontal). The coordinates of the point A are $L=\sqrt{3}mcr_{s}$ and $E_{0}=\sqrt{\frac{8}{9}}mc^{2}$. The innermost unstable circular orbit occurs at $r=1.5r_{s}$ for infinite values of L and $E_{0}$—marked in the figure by a brown dashed horizontal asymptote. Beneath the innermost stable circular orbit, neither a circular nor any local closed orbit exists. The event horizon and the photon sphere rim (the innermost unstable circular orbit) are shown for illustration.

Using Equation (5), one can write out the conditions for circular orbits, $U\left(r\right)=E_{0}$, in the extremes of the potential, $\frac{\partial U(r)}{\partial r}=0$, in the following form [36]:

(6) $$\begin{array}{c} E_{0}=Lc\sqrt{\frac{2}{rr_{s}}}\left(1-\frac{r_{s}}{r}\right),\\ \frac{r}{r_{s}}=\frac{L^{2}}{m^{2}c^{2}r_{s}^{2}}\left[1\pm \sqrt{1-\frac{3m^{2}c^{2}r_{s}^{2}}{L^{2}}}\right], \end{array}$$

where the sign + in the second equation corresponds to stable orbits (at the minimum of U(r)) and the sign − to unstable ones (at the maximum of U(r)). Particular stable and unstable circular orbits depend on the angular momentum L and on the energy $E_{0}$ of a particle. In Figure 2, the radii of stable and unstable circular orbits of the particle in the vicinity of the event horizon are shown as functions of the angular momentum L, which are determined by the solution of the second equation in the pair (6)—for sign +, the upper branch (the blue line in the figure) gives the radii of stable circular orbits. This branch terminates in the point A at $r=3r_{s}$—i.e., the innermost stable circular orbit exists at $r=3r_{s}$ for $L=\sqrt{3}mcr_{s}$ and $E_{0}=\sqrt{\frac{8}{9}}mc^{2}$ (the energy is determined by the first equation of the pair (6)). The innermost unstable circular orbit (in the solution of the second equation of (6) with the sign −) occurs at $r=1.5r_{s}$ for $L,E_{0}\to \infty$. The branch for unstable circular orbits (marked by red line in the figure) begins at point A and terminates asymptotically in infinity at the radius $1.5r_{s}$. Below this radius, no circular orbit exists. Though Equation (6) is written for a particle with mass *m*, the above derivation can be repeated for zero rest mass particles (like photons). The innermost unstable circular orbits occurs at $r=1.5r_{s}$ for all particles regardless of their mass (also for photons with zero rest mass). This universal limiting orbit is frequently called the photon sphere rim (the photon sphere extends from the rim to the event horizon).

**Figure 3:**
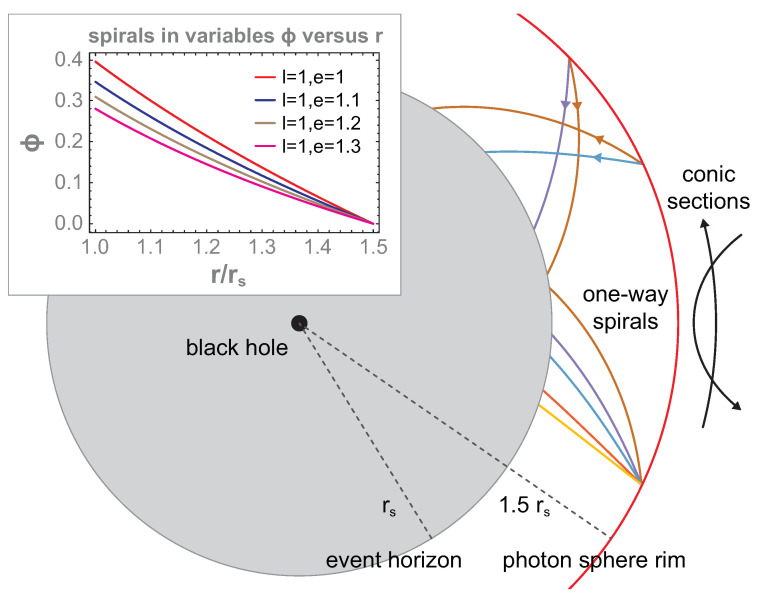
Trajectories of particles which have crossed the photon sphere rim inward, have the form of short spirals directed onto the event horizon—this follows from the solution of Equation (3) in the radius sector $r\in (r_{s},1.5r_{s})$. In the figure, there are shown these spirals for several initial conditions and motion integrals L and $E_{0}$ as specified in the Inset (in which spirals are shown in the coordinates—azimuthal angle versus radius). Though unidirectional spirals can mutually intersect (for opposite signs of angular momenta), it is impossible to close any loop built from their pieces. It means that particles in the photon sphere cannot mutually exchange positions if they belong to the multi-particle system which has passed the photon sphere rim inward. The timing of traversing these spirals is defined by Equation (2) written in the ordinary time of a remote observer. Changing to the proper time (or to any other curvilinear coordinates in different metrics) does not change the homotopy class of these spirals—local closed loops are not admissible beneath the photon sphere rim in contrary to the upper neighborhood, where arbitrary small local loops are possible due to the crossing of conic sections.

The even more important property of the photon sphere rim (the innermost unstable circular orbit) is the unavoidable falling of any particle toward the event horizon if it was passing the photon sphere rim inward. This property follows directly from the motion of Equations (2) and (3). They describe a specific motion of particles in the region $r_{s}<r<1.5r_{s}$ in the form of unidirectional short spirals directed toward the event horizon for any particle passing the photon sphere rim inward. These spirals are exemplified in Figure 3 for several values of motion integrals L and $E_{0}$ ($l=\frac{L}{mcr_{s}}$ and $e=\frac{E_{0}}{mc^{2}}$). The reverse traveling of particles is also possible (which means the escape of a particle from the photon sphere), but this requires a change in the initial conditions (to inverse velocity) unavailable for free particles falling from the outside across the photon sphere rim as is considered here. All particles entering the photon sphere from outside must fall unavoidably toward the event horizon—their movement is locally unidirectional—as is visualized in Figure 3. Falling particles achieve the event horizon at t=∞ as is noticeable from Equation (2) (thus, the event horizon in non-observable for any remote observer), but if one changes to the proper time, then the event horizon is passing by falling particles at finite proper time τ and the whole dynamics of these particles finishes in a central singularity of a black hole also after a finite τ period. However, below the event horizon, any change in the initial conditions does not allow particles to escape this region. The event horizon is an ostensible singularity (visible in the Schwarzschild metric (1) at $r=r_{s}$) and can be removed by changing to non-stationary or non-rigid coordinates as was demonstrated by various redefinitions of the metric, e.g., [37,38,39]. This arbitrariness in the description of the same folded spacetime follows from the freedom in the selection of time coordinates versus space ones (corresponding to the various slicing of the same folded spacetime into spatial and temporal pieces). In particular, the Schwarzschild metric (1) is suited to properly describing the outer region with respect to the event horizon in the rigid stationary coordinates, the same as a remote observer. These coordinates are especially convenient to analyze in a transparent manner the homotopy of trajectories of free particles falling onto the event horizon from outside of the photon sphere, though the homotopy of these trajectories is the same in any other coordinates (regardless of the metric choice).

Using Equations (2) and (3), one can analyze the trajectory behavior in various sectors of the event horizon vicinity. For $r>1.5r_{s}$, any free-particle trajectories have the shape of conic-like sections. Distinct from conic sections for Newton-type gravitational singularity, in Schwarzschild geometry, some additional precession of ellipse occurs (like of Mercury in the sun’s gravitation), which does not, however, change the homotopy class of trajectories in this region. Conic-like sections can have both opposite orientations of the particle movement. In addition, there always exist two distinct and oppositely oriented conic-like sections, which can mutually cross in two different closely located points, creating local arbitrarily small closed loops (like an ellipse intersecting with a circle or hyperbole, and so on). Such a possibility defines the trajectory homotopy class of these trajectories for multi-particle systems beyond the photon sphere rim. In cross-points of two oppositely oriented conic section-like trajectories, two different particles can be placed, and the local loop built of these conic-like section pieces displays the exchange of these particles—in other words, exchanges of particle pairs are possible beyond the photon sphere rim.

Nevertheless, beneath the photon sphere rim for $r_{s}<r<1.5r_{r}$, Equations (2) and (3) do not describe conic-like sections any more, but define only short spirals as admissible free particle trajectories—as illustrated in Figure 3. These spirals are unidirectional for all particles in any local multi-particle system passing the photon sphere rim inward. From pieces of these unidirectional spirals, it is impossible to create a local closed loop. Beneath the photon sphere rim, there do not exist local closed loops for mutual particle exchanges because it is impossible to build such loops from even an arbitrarily large number of pieces of one-way spirals approaching the event horizon. This is a specific property of general relativistic gravitational singularity and is easily noticeable in the Schwarzschild metric, which properly describes the upper neighborhood of the event horizon in the coordinates of a remote observer. For such an observer, the time *t* needed to approach the event horizon by any particle is infinite, but the trajectory shape (and a related homotopy class of trajectories) is independent of the motion rate. The homotopy class is the same if one changes to the proper time τ, which allows to describe the passing of a particle by the event horizon within a finite period of the proper time and the termination of a particle movement in the central singularity also after a finite proper time period. This can be illustrated in non-stationary metrics, e.g., by Kruskal–Szekeres [37,38] or in metrics by Novikov [39]. The homotopy of trajectories is the same in any coordinates (is immune to the deformation), but the selection of the Schwarzschild metric (1) is convenient to analyze trajectories without a need to modify Feynman path integrals written in stationary and rigid coordinates of a remote observer (cf. Appendix A) convenient to quantize classical multi-particle systems and to identify quantum statistics. The Schwarzschild metric (1), similar to other metrics, displays the same gravitationally folded spacetime, and various metrics correspond only to a different slicing of the same curved spacetime into its spatial and temporal components. This gravitationally curved spacetime has an inherent property, which is that it is not admitted a description in stationary rigid spatial coordinates simultaneously with the inner and outer regions with respect to the event horizon [36]), and the Schwarzschild metric expressed in time-independent (stationary) spatial coordinates, the same as of a remote observer, is suited to the upper vicinity of the event horizon.

Below the photon sphere rim for $r\in (r_{s},1.5r_{s})$, no closed local loops built of pieces of one-way short spirals given by Equations (2) and (3) exist (cf. Figure 3), whereas above this rim ($r>1.5r_{s}$), trajectories are of the conic section type, allowing arbitrarily small closed loops built of trajectory pieces. This is a qualitative change in the homotopy class of trajectories in multi-particle systems of free indistinguishable identical particles when passing the photon sphere rim.

The change in the homotopy class for particle classical trajectories in multi-particle systems of identical indistinguishable particles described above is of fundamental significance for the quantization of such multi-particle systems and for the assigning of a quantum statistics to particles in these systems. Quantum statistics is topologically conditioned [27,32,40], and is assigned to identical indistinguishable particles according to a scalar unitary representation of a braid group [25] describing all accessible exchanges of positions of these particles. Usually, braid groups have multiple scalar unitary representations defining different quantum statistics for the same classical particle archetype. The braid group is the first homotopy group $\pi_{1}$ of the multi-particle configuration space of a system [23,25,26]. For *N* identical indistinguishable particles placed on some manifold M, the configuration space $F_{N}$ is defined as follows:

(7) $$F_{N}=\left(M^{N}-\Delta \right)/S_{N},$$

where $M^{N}=M\times M\times \cdots \times M$ is the *N*-fold product of the manifold M to account for positions of all particles equally, Δ is a diagonal subset of $M^{N}$, which contains points with at least two particle coordinates coinciding. Δ is removed from the coordination space in order to assure the particle number conservation. The division of the space by the permutation group $S_{N}$ introduces the indistinguishability of particles and according to the definition of the quotient space, points in $F_{N}$, which differ in particle numbering, only are unified into a single point. The braid group is defined as $\pi_{1}\left(F_{N}\right)$ (the first homotopy group [23,25,26]) and according to the definition, it collects non-homotopic classes of closed loops in $F_{N}$. Loops from different non-homotopic classes cannot be deformed in a continuous manner, one into another, without cutting. Loops from $\pi_{1}\left(F_{N}\right)$ are multi-strand trajectories in $F_{N}$, joining points with different numbers of particles (unified into a single point in $F_{N}$) and thus describe all possible interchanges of particle positions on M. The braid group $\pi_{1}\left(F_{N}\right)$ is a multi-cyclic group generated by a finite set of generators, $\left\{\sigma_{i},\;i=1,\cdots ,N-1\right\}$, which are elementary braids—$\sigma_{i}$ is a braid corresponding to the exchange of the position of the *i*-th particle with the (i+1)-th one when other particles stay in rest (numeration of particles is arbitrary but fixed) [25,26]. For 3D spatial manifolds in unfolded spacetime, $\sigma_{i}^{2}=e$ (neutral group element) for all *i*; hence, for three-dimensional manifolds, $\pi_{1}\left(F_{N}\right)=S_{N}$ independently of a manifold form [25,26]. There exist only two different scalar unitary representations of $S_{N}$ defined on generators, $\sigma_{i}\to e^{i\pi }\;or\;e^{i0}$, corresponding to fermions and bosons, respectively. They correspond to two different quantization ways of the same classical particles on 3D manifolds.

In the Schrödinger quantum mechanics representation, the multi-particle wave functions must transform according to a scalar unitary representation of the braid when wave function coordinates (classical positions of particles on a manifold M) exchange in a way defined by this particular braid. As for 3D manifolds, braids are permutations only, and thus multi-particle wave functions must be antisymmetric for fermions and symmetric for bosons. Note, that even for free particles, the multi-particle wave functions for fermions as well for bosons are quantumly entangled states corresponding to non-separable functions in *N*-particle tensor productive Hilbert space. This entanglement is not induced by any interaction.

For 2D spatial manifolds, braid groups are different from $S_{N}$. In the 2D case, $\sigma_{i}^{2}\neq e$, and braid groups are infinite (but countable, as the generator set $\left\{\sigma_{i},\;i=1,\ldots ,N-1\right\}$ is finite). For $M=R^{2}$ (a plane), the braid group is the infinite Artin group [26,30]. The Artin group has also an infinite number of different scalar unitary representations $\sigma_{i}\to e^{i\alpha }$ with α∈[0,2π). Each α defines a different sort of quantum particle—so-called anyons [31]. Anyons satisfy fractional quantum statistics.

Topological constraints imposed on multi-particle trajectories affect quantum statistics (as exemplified above in the case of a two-dimensional manifold defining a collective system). Specific homotopy restrictions for trajectories close to a gravitation singularity in spacetime folded along the general relativity Einstein’s equations also modify quantum statistics for particles on any local manifold in the vicinity of the event horizon.

### 2.2. Quantum Statistics Transition in Multi-Particle Systems Passing the Photon Sphere Rim of a Black Hole

The formal mathematical definition of the quantum statistics (valid also in the relativistic case) can be formulated within the quantization by Feynman path integration [33,34] extended for multi-particle systems of identical indistinguishable particles [27,41,42]. A path integral for *N* indistinguishable identical particles has the form [27,41,42,43]

(8) $$\begin{array}{c} I(Z_{1},t_{1};Z_{2},t_{2})\\ =\sum_{l\in \pi_{1}}e^{i\alpha_{l}}\int d\lambda_{l}e^{iS[\lambda_{l}\left(Z_{1},t_{1};Z_{2},t_{2}\right)]/\hbar }, \end{array}$$

where points $Z_{1}=\left(z_{1}^{1},\ldots ,z_{N}^{1}\right)$ and $Z_{2}=\left(z_{1}^{2},\ldots ,z_{N}^{2}\right)$ are two different points in multi-dimensional configuration space $F_{N}$ that define the start and final points for the propagator $I(Z_{1},t_{1};Z_{2},t_{2})$ between time instants $t_{1}$ and $t_{2}$, respectively. This propagator is the matrix element of the quantum evolution operator for a whole system in the position representation (between localized states characterized by particle positions $Z_{1}$ and $Z_{2}$), i.e., it is a complex amplitude of the probability for quantum transition between these localized states. $S[\lambda_{l}\left(Z_{1},t_{1};Z_{2},t_{2}\right)]$ in Equation (8) is the classical action, i.e., the time integral of the Lagrangian of the whole system along the trajectory $\lambda_{l}\left(Z_{1},t_{1};Z_{2},t_{2}\right)$—this is a functional over the path space. In the Lagrangian, an interaction can be included between particles.

To an open trajectory linking $Z_{1}$ and $Z_{2}$ in $F_{N}$, an arbitrary braid loop can be attached to an arbitrary intermediate trajectory point, which reflects the possibility of particle numbering changes on the way (this is due to particle indistinguishability). The discrete index *l* in Equation (8) numerates braids in the full braid group $\pi_{1}\left(F_{N}\right)$, and $e^{i\alpha_{l}}$, $\alpha_{l}\in \left[0,2\pi \right)$ is the scalar unitary representation of the *l*-th braid (braid groups are countable; thus, *l* is discrete). Different braids are non-homotopic (cannot be deformed in a continuous manner one into another without cutting); thus, trajectories with attached braids split into non-homotopic families without any continuous linkage. This precludes the definition of the measure in the whole path space (because of the absence of the continuity between sectors), and therefore, the measure in the path space can be only defined separately on each disjoint sector of this space numbered by *l*—the resultant family of measures for path integration is denoted by $d\lambda_{l}$ in (8). The contributions to the path integral from all sectors of the path domain numbered by braid group elements must be finally added with an arbitrary unitary phase factor (unitarity is required to be consistent with the quantum mechanics causality). These unitary factors form scalar unitary representations of the full braid group [27]. The arbitrariness of phase factors translates into the multiplicity of scalar unitary representations of a particular braid group [27]. Any representation assigns different quantum statistics for particles on manifold M [25]. For 3D manifolds in unfolded space, the braid group is always equal to the permutation group $S_{N}$, which has two different scalar unitary representations, $\sigma_{i}\to \pm 1$, assigning bosons and fermions, respectively. For 2D manifolds, the braid groups are different from $S_{N}$ and have more scalar unitary representations assigning anyons besides bosons and fermions [31].

The braids attached to an open multi-strand trajectory in $F_{N}$ must be built of pieces of classically accessible trajectories, similarly to all paths for the Feynman integral over trajectories [34,43]. The trajectories entering the Feynman path integral must belong to the domain of the measure in the path space. This measure is constructed in analogy to the Wiener measure [44] in path integrals for stochastic processes used earlier to describe Brownian motion [41] and next extended by Feynman for quantum path integrals (with the difference resolving itself to a complex integrand in comparison to the Wiener integral with a real integrand). The requirements imposed on the domain of the measure in the path space are the same for both types of integrals [41] and specify what ’summation over all paths’ means in (8). In fact, not all paths contribute but only those which are congruent with the measure definition (i.e., belong to the measure domain). The definition of the measure in the Feynman path integral is the same as that for the Wiener measure [44] and arises via the time discretization and the creation of piece-wise continuous (but not smooth, in general) paths with fixed start and final points but with arbitrary positions of intermediate points joining consecutive sectors of the time discretization. Here, a restriction occurs, however—trajectory pieces on each sector of time discretization must exist as classical attainable trajectories; otherwise, the construction of the path is ineffective, and the corresponding path must be discarded from the path domain. Such situations have been encountered in Brownian motions with barriers and noticed for quantum path integrals by Pauli [41] (for more details and summary, cf. Appendix A).

In the exceptional situation where classical trajectories for particle position exchanges do not exist on intermediate segments of the time discretization, it is impossible to define braids except for a trivial one *e* (neutral group element without any path). In such a case, the summation over *l* in Equation (8) disappears, and no quantum statistics can be assigned. We encounter such restrictions in the case of any multi-particle system beneath the photon sphere rim of a black hole.

In the case of the trajectory homotopy class occurring in the vicinity of the event horizon of a black hole, for multi-particle systems of free particles passing inward from the photon sphere rim, braids cannot be constructed from pieces of unidirectional spirals defined by Equations (2) and (3) for $r\in (r_{s},1.5r_{s})$ because local loops cannot be closed here by any number of one-way trajectory pieces (cf. Figure 3). For particles in the multi-particle system that pass the photon sphere rim inward, no braid can be constructed except for a trivial braid *e*. The multi-particle configuration space is thus simply connected here (the term simple connectivity is addressed in the case of a space where the first homotopy group $\pi_{1}$ of this space is a trivial group {e}) and no quantum statistics can be assigned to particles beneath the photon sphere rim [43,45]. If $\pi_{1}\left(F_{N}\right)=\left\{e\right\}$, then the sum over *l* in Equation (8) disappears, and scalar unitary representation is only e→1 (because e·e=e, and it holds also for any representation), and this representation does not assign any statistics, as *e* does not define any particle exchange.

This is different compared to the Newton gravitation center, for which conic section trajectories and local arbitrarily small loops for particle exchanges are available in arbitrary close vicinity to the point-like gravitational center. For the Newton gravitational center various, conic sections that are oppositely directed can cross at arbitrarily close points, making it possible to create closed small local loops describing particle mutual interchanges. Even though for a large distance from the gravitational general relativistic center, the Schwarzschild trajectories can be approximated by conic section-like curves (modified by some additional deformation, e.g., a precession of elliptical orbits, similar to that observed for Mercury in the sun’s gravitation), in close vicinity to the event horizon, conic section-like trajectories completely disappear below the photon sphere rim. The one-way spirals that are accessible beneath the photon sphere rim (described by Equations (2) and (3) as visualized in Figure 3 for $r_{s}<r<1.5r_{s}$) do not allow the closing of a small local loop built of even an arbitrary large number of pieces of these unidirectional spirals. Hence, for $r\geq 1.5r_{s}$, the fermionic or bosonic quantum statistics can be assigned [25,26] in contrary to the region $r\in (r_{s},1.5r_{s})$, where no quantum statistics is defined [43]. In this region, the Pauli exclusion principle is locally waived off, in particular. Though geodesics defined by Equations (2) and (3) describe the trajectories of free particles in multi-particle systems, such systems are sufficient to define quantum statistics, as the local deformations due to interaction between particles cannot change the homotopy class of their trajectories.

## 3. Results

### 3.1. Collapse of Fermi Spheres in Dense Systems of Fermions Passing the Photon Sphere Rim

**Figure 4:**
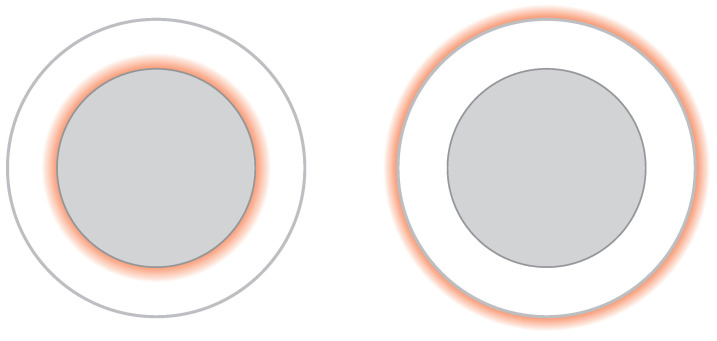
Illustrative drawing of the shift of a firewall from the event horizon to the photon sphere rim. The firewall on the event horizon proposed by Polchinski [6] in order to cope with the information paradox would be invisible for any remote observer. The decay of Fermi spheres in compressed matter passing the photon sphere rim is the source of intensive e-m radiation emission, which takes away the energy and entropy of matter consumed by a black hole—thus, it can take the role of the firewall, considerably changing the premises of the information paradox. Such a firewall would be visible to any remote observer—in particular, it can be responsible for some giant gamma-ray bursts associated with collapses of neutron star mergers or large non-thermal radiation of superluminous quasars.

The local decay of quantum statistics (described above) is associated with the release of energy upon passing inward from the photon sphere rim by any multi-particle macroscopic system of fermions structured due to the Pauli exclusion principle. This energy emission in the form of electromagnetic radiation according to the Fermi golden rule admitted here for quantum transitions (due to the local departure from the Pauli exclusion principle, which outside the photon sphere blocks such transitions) takes away the entropy along with the energy, and the particles devoid of quantum statistics create a pure quantum state of individual particles which cannot interchange their positions, like in an ideal crystal invoked in the third law of thermodynamics by Nernst. The matter that next crosses (within a finite length of the proper time) the event horizon does not carry the entropy or information related to quantum statistics, which modifies premises for the information paradox [1,2] and the firewall concept at the event horizon of a black hole [6]. The role of some kind firewall is taken by the, visible to any distant observer, radiation burst released during the passing by the falling matter of the photon sphere rim relatively distantly from the event horizon. The shift of the firewall to the photon sphere rim is schematically shown in Figure 4.

Each multi-particle system which passes the photon sphere rim inward loses its quantum statistics. This does not violate the Pauli theorem on spin and statistics as shown in Appendix B.

The exclusion principle for fermions asserts that quantum particles of the fermionic type cannot share any common single-particle quantum state. In particular, fermions cannot share the same localized single-particle quantum states, which manifest themselves when approaching, by a fermion, a space region already occupied by another one. Hence, identical indistinguishable fermions repulse themselves mutually. This is addressed as the quantum degeneracy repulsion, and in multi-fermion systems, it raises a pressure related to this repulsion (without contribution of any elementary interaction forces). This pressure can exert giant strength forces which are able to stop the collapse of white dwarfs (due to the quantum degeneracy repulsion of electrons) [46] or neutron stars (where a gravitation collapse is halted by the quantum degeneracy repulsion of neutrons) [16,17,47].

The exclusion principle for fermions leads also to the formation of a Fermi sphere in the case of a large number of identical fermions located in some volume; if the chemical potential μ (the energy increase in a thermodynamic multi-particle system caused by the addition of a single particle to the system) is much greater than the temperature *T* of the system expressed in an energy scale $\mu \gg k_{B}T$, then $k_{B}$ is the Boltzmann constant. In such systems, fermions are forced to occupy consecutive energy single-particle stationary states one by one, resulting in the accumulation of energy because the ladder of consecutive filled energy states is as large as the number of particles in the system. In an isotropic case, when the stationary states are numbered by momentum |p| (like for free particles with energy $\frac{p^{2}}{2m}$ or relativistic ones $\sqrt{c^{2}p^{2}+m^{2}c^{4}}-mc^{2}$), the filled states form a sphere in momentum space with a finite radius called the Fermi momentum $p_{F}$. Such a collective state in a multi-particle system of fermions accumulates a great amount of energy in the Fermi sphere (greater the higher the density of fermions). For example, free electrons in metal under normal conditions with a typical concentration of the Avogadro number per cm^3^ accumulate in a free electron Fermi sphere an energy of the order of $10^{10}$ J/m^3^. In a neutron star with a mass of 2.3 sun masses compressed to a sphere with a radius of the order of 6–10 km, the Fermi sphere of neutrons accumulates ca. $10^{46}$–$10^{47}$ J. When the Fermi statistics is defined (as outside of the black hole photon sphere), the Fermi spheres in multi-particle systems are stable, and the stored energy cannot be released because of the blockade by the Pauli exclusion principle. The situation changes, however, when quantum statistics cannot be defined below the photon sphere rim of a black hole. The energy accumulated in the Fermi sphere of strongly compressed fermion systems passing the photon sphere rim inward can be released here.

Fermi momentum $p_{F}$, i.e., the radius of the Fermi sphere in the degenerate homogeneous quantum liquid of fermions, is a function of the particle concentration solely [48]:

(9) $$p_{F}=\hbar {\left(3\pi^{2}\rho \right)}^{1/3},$$

where $\hbar =\frac{h}{2\pi }$ is the reduced Planck constant, and $\rho =\frac{N}{V}$ is the concentration of *N* fermions in the spatial volume *V*. Remarkably, the Fermi momentum is independent of the interaction of fermions according to the Luttinger theorem [48,49]. This fact can be noticed by quasiclassical reasoning and is thus immune to interaction. According to the Bohr–Sommerfeld rule, the phase space equivalent to the Fermi sphere volume $\frac{4}{3}\pi p_{F}^{3}$ in momentum space and the spatial volume *V* corresponds to $\frac{2V4\pi p_{F}^{3}}{3{\left(\hbar 2\pi \right)}^{3}}$ single-particle quantum states (the additional 2 in the numerator is due to the spin degeneracy for fermions with $\frac{1}{2}$ spin). If all these states are filled by *N* particles, then Formula (9) is reproduced.

The whole Fermi sphere gathers the energy in spatial volume *V* (neglecting the interaction of fermions):

(10) $$\begin{array}{c} E=\sum_{p}\varepsilon \left(p\right)f\left(\varepsilon \left(p\right)\right)\\ =\frac{V}{{\left(2\pi \hbar \right)}^{3}}\int d^{3}p\varepsilon \left(p\right)f\left(\varepsilon \left(p\right)\right)\\ =\int_{0}^{p_{F}}dp\int_{0}^{\pi }d\theta \int_{0}^{2\pi }d\phi p^{2}sin\theta \varepsilon \left(p\right)\frac{V}{{\left(2\pi \hbar \right)}^{3}}\\ =\frac{V}{2\pi^{2}\hbar^{3}}\int_{0}^{p_{F}}dpp^{2}\varepsilon \left(p\right), \end{array}$$

where the sum runs over occupied states only, which is assured by the Fermi–Dirac distribution function $f\left(\varepsilon \left(p\right)\right)=\frac{1}{e^{\left(\varepsilon \left(p\right)-\mu \right)/k_{B}T}+1}\to_{T\to 0}1-\Theta \left(\varepsilon \left(p\right)-\varepsilon_{F}\right)$ (Θ(x) is the Heaviside step function, $\varepsilon \left(p\right)=\sqrt{p^{2}c^{2}+m^{2}c^{4}}-mc^{2}$ (in the relativistic case), $\varepsilon_{F}=\varepsilon \left(p_{F}\right)=\mu \left(T=0\right)$ is called Fermi energy, and μ is the chemical potential at T=0), and p,θ,ϕ are spherical variables in the momentum space. The energy estimation Equation (10) holds for zero temperature, as well as for non-zero temperatures, if $k_{B}T\ll \mu ≃\varepsilon_{F}$ (i.e., when the Fermi liquid is quantumly degenerated).

For a neutron star at the TOV limit [16,17] with the density of the order of $5\times 10^{18}$ kg/m^3^ (corresponding to 2.3 sun masses compressed to a compact neutron star with a radius of ca. 6 km), the neutron Fermi sphere energy reaches $0.5\times 10^{47}$ J (cf. Table 1), just like the energy of the frequently observed cosmic short giant gamma-ray bursts (assuming the isotropy of their sources). The Fermi energy in this case is $\varepsilon_{F}≃0.34$ GeV (i.e., $≃4\times 10^{12}$ K in temperature units, when $k_{B}=1$ is assumed), which is much greater than the supposed temperature of a neutron star, of the order of $10^{6}$ K (thus, neutrons in a neutron star form a degenerate quantum system).

The coincidence of the energy stored in the Fermi sphere of neutrons in a neutron star at TOV limit with the energy of short giant gamma-ray bursts supports the idea that the source (yet unknown) of some of these bursts is a collapse of the Fermi sphere of neutrons when the whole star is compressed to the volume inside its own photon sphere. The assistance of the collapses of neutron star mergers crossing the TOV limit by giant γ-ray bursts was suspected earlier, but the mechanism of the conversion of ca. 0.25 sun masses into electromagnetic radiation within a subsecond time was beyond any earlier known mechanisms. The appropriate mechanism is, however, offered by the local decay of quantum statistics. During the related decay of the Fermi sphere of neutrons, the latter, liberated from the Pauli exclusion principle constraint, falls apart when charged electrons and protons interact with the electromagnetic field.The jumping of these particles onto their ground state upon the decay of the neutron Fermi sphere will release a giant flux of isotropic electromagnetic radiation along the Fermi golden rule for quantum transitions, with a dominant component of the gamma radiation because of the large value of Fermi energy in this case, in agreement with the spectral features of observable short gamma-ray bursts (cf. Table 1 and Table 2).

In another example, in an accretion disc of a quasar, the density of electron and proton plasma (assuming the accretion of neutral hydrogen) grows with the falling of the matter toward the event horizon. The diluted neutral gas is ionized in the accretion disc due to friction and eventually becomes a dense plasma of electrons and protons, both of which are degenerate Fermi liquids. Even though the local temperature in the accretion disk can be high (even up to $10^{6}$ K in the vastaccretion disc of large quasars), the Fermi energy of electrons and of protons at the photon sphere rim is of the order of GeV (i.e., of the order of $10^{13}$ K), which means that both components of plasma are quantumly degenerate liquids. For two-component plasma in the accretion disc of the quasar, both Fermi spheres of electrons and protons contribute to the energy storage. This energy grows at the cost of the gravitational energy of the central black hole, which compresses multi-particle systems to an extremely high concentration, increasing the Fermi sphere size. At the same concentration of electrons and protons (due to the neutrality of plasma in the disc at accretion of a neutral gas), electrons accumulate larger kinetic energy than protons because of the lower electron rest mass. The energy accumulated in the Fermi spheres of electrons and protons can be released in the form of the electromagnetic radiation if the Pauli exclusion principle is locally waived off at the rim of the photon sphere, due to the local decay of quantum statistics. In the case of steady conditions at the matter accretion by a black hole, the continuous-in-time process of extracting energy from a stable flux of plasma takes place, and the electromagnetic radiation can be counted per second, resulting in stable contribution to the total luminosity of a quasar expressed in J/s. The source of this radiation is located at the photon sphere rim at a distance of $1.5r_{r}$ from the singularity center. This is far below the accretion disc inner edge assumed in the conventional classical hydrodynamic models of these discs [19,39,50]; thus, this non-thermal radiation does not conflict with the radiation from more distant regions of the accretion disc. Taking into account the high luminosity of the radiation associated with the Fermi sphere decay, it can significantly contribute to the total luminosity from the accretion disc and solve the long-standing problem of the discrepancy of conventional models [19] with observations of superluminous quasars [20,21,51]—for more details and comparison with observations, cf. Section 4.2.

Superluminous quasars with a central black hole ∼$10^{9}$ $M_{⊙}$ consume typically 10 sun masses per year (0.1 Earth mass per second), when the accretion of the gas is limited only by the uppermost density of stable matter at the photon sphere rim (similar to the density of neutron star at TOV limit or to the density of atom nuclei). The continuous decay of Fermi spheres of electrons and protons in accretion plasma crossing the photon sphere rim of a supermassive central black hole releases photons with a total energy of up to 30% of the falling mass [22,45]. This fraction of the mass is the energy (divided by $c^{2}$) of the Fermi spheres of electrons and protons accumulated at the cost of the black hole gravitation during matter compression in the accretion disc. This rapidly released energy, in the form of electromagnetic radiation, contributes to the luminosity of superluminous quasars with ca. $10^{40}$ W at a close vicinity to the event horizon (when passing the rim of the photon sphere at $r=1.5r_{s}$), in better agreement with the observations than only radiation from more distant regions of the accretion disc [19,20,21,52]. If the supply of the matter to the accretion disc is limited by environmental conditions, then the density of plasma at the photon sphere rim is not extreme, and the efficiency of the Fermi sphere decay is much lower than 30% (cf. Table 3). The detailed quantitative estimations, including general relativistic corrections of the density and a comparison with examples of astrophysical observations, are presented in Section 4.2.

### 3.2. Spectrum and Timing of Short Giant Gamma-Ray Burst at Unstable Neutron Star Merger Collapse

A rapid departure from the Pauli exclusion principle relieves the internal quantum degeneracy pressure in the neutron star merger, allowing it to collapse (when the merger is compressed by its own gravity to the volume beneath the photon sphere rim of the corresponding black hole). The decay of the Fermi sphere of fermions releases energy accumulated in this sphere in the form of electromagnetic radiation along the Fermi golden rule for quantum transitions of charged particles (products of the decomposition of neutrons) admitted when the matter compression is beyond the TOV limit (when the whole neutron star is inside its own photon sphere). The energy of the neutron Fermi sphere in the neutron star merger at the TOV limit largely agrees with the energy of the observed giant short gamma-ray bursts of total energy ∼$10^{46}$–$10^{47}$ J (assuming isotropic their sources). Free neutrons are unstable with the time decay for electrons, protons, and electron antineutrinos of the order of 15 min. In neutron stars, neutrons are stabilized only by the Pauli exclusion principle (similarly to stable nuclei). When the quantum statistics decays, the neutrons decompose into stable electrons and protons—charged particles interact with an electromagnetic radiation and neutral antineutrino, along $\beta^{-}$ decay exponentially accelerated by large number of neutrons in a neutron star merger (resulting in a collective avalanche-type $\beta^{-}$ transition governed by the Fermi golden rule). Liberated charged electrons and protons interact with an electromagnetic field and rapidly jump onto lower quantum states emitting photons. The kinetics of these transitions can be assessed also by the application of the Fermi golden rule.

This rule gives the probability per time unit of quantum transitions between two stationary quantum states of a charged particle induced by the time-dependent perturbation of an electromagnetic field. For an initial quantum stationary state |1〉 of a particle with energy $E_{1}$ and final one |2〉 with energy $E_{2}$, this transition probability is given by the following expression [53]:

(11) $$w_{1,2}=\frac{2\pi }{\hbar }\left|\langle 1\right|\hat{V}{\left(r\right)|2\rangle |}^{2}\delta \left(E_{1}-E_{2}-\alpha \hbar \omega \right),$$

where $\langle 1|\hat{V}\left(r\right)|2\rangle$ is the matrix element of an operator $\hat{V}\left(r\right)$ describing the coupling of a charged particle to the electromagnetic field taken between the initial and final states, and αℏω is the energy of an emitted photon. The Dirac delta expresses the energy conservation at this transition. As we consider the transition close to the black hole event horizon at the photon sphere rim, where it is admitted due to the local departure from the Pauli exclusion principle, a gravitational redshift of photon frequency $\alpha ={\left(1-\frac{r_{s}}{r}\right)}^{1/2}≃0.57$ for $r=1.5r_{s}$ is included. Relativistic electrons or protons interacting with e-m field have single-particle Hamiltonians, ${\hat{H}}_{e(p)}=\sqrt{{\left(\hat{p}\mp eA\left(r,t\right)\right)}^{2}c^{2}+m_{e(p)}^{2}c^{4}}-m_{e(p)}c^{2}$, where $A\left(r,t\right)=A_{0}e^{i(q\cdot r-cqt)/\hbar }$ is the vector potential of the e-m field (of the plane wave form, at the chosen gauge divA=0) and $\hat{p}=-i\hbar \nabla$. The Fermi golden rule describes quantum transitions with linear accuracy with respect to the perturbation [53]; therefore, the operator $\hat{V}$ in Equation (11) must be taken as the term that is linear with respect to A in the Hamiltonian, which is as follows:

(12) $$\hat{V}\left(r,t\right)=\mp \frac{ec^{2}A\left(r,t\right)\cdot \hat{p}}{\sqrt{{\hat{p}}^{2}c^{2}+m_{e(p)}^{2}c^{4}}},$$

where $e=1.6\times 10^{-19}$ C, and ± corresponds to an electron and a proton, respectively.

For isotropic systems with local translational symmetry, the states |1(2)〉 can be taken in the form $|1\left(2\right)\rangle =\frac{1}{{\left(2\pi \hbar \right)}^{3/2}}e^{i(p_{1(2)}\cdot r-E_{p_{1(2)}}t)/\hbar }$, i.e., states in the Fermi sphere with $p_{1(2)}\leq p_{F}$, $p_{1}>p_{2}$. The matrix element in Equation (11) can be calculated analytically, and it gains the form

(13) $$\langle p_{1}\left|V\left(r\right)\right|p_{2}\rangle =\mp \delta \left(p_{1}-p_{2}-q\right)\frac{ec^{2}A_{0}\cdot p_{2}}{\sqrt{p_{2}^{2}c^{2}+m_{e(p)}^{2}c^{4}}}.$$

Equation (11) can be thus rewritten as follows:

(14) $$\begin{array}{c} w_{1,2}=\frac{V}{{\left(2\pi \right)}^{2}\hbar^{4}}e^{2}c^{2}A_{0}^{2}cos^{2}\theta f\left(p_{2}\right)\delta \left(p_{1}-p_{2}-q\right)\\ \times \delta (E_{p_{1}}-E_{p_{2}}-\alpha cq), \end{array}$$

with $f\left(p_{2}\right)=\frac{p_{2}^{2}c^{2}}{p_{2}^{2}c^{2}+m_{e(p)}^{2}c^{4}}<1$, and θ is the angle between vectors $A_{0}$ and $p_{2}$ (for details of the calculation, in particular, the way of treatment with the Dirac delta square, cf. [15]).

Next, the integration of Equation (14) over all initial and final states in the Fermi sphere (up to $p_{F}$) must be performed to estimate the time span for an entire Fermi sphere decay. This integration over $p_{1}$ and $p_{2}$ gives the result:

(15) $$\begin{array}{c} w=\int_{p_{F}}d^{3}p_{1}\int_{p_{F}}d^{3}p_{2}w_{1,2}=\left(N+1\right)\gamma ,\\ \gamma =\frac{mce^{2}}{3\pi \varepsilon_{0}\hbar^{2}y}\int_{-1}^{1}dz\int_{0}^{p_{F}/mc}dx\frac{x^{4}}{x^{2}+1}\\ \times \delta (\sqrt{x^{2}+y^{2}+2xyz+1}-\sqrt{x^{2}+1}-0.57y), \end{array}$$

where $x=p_{2}/mc$, $y=q/mc=\hbar \omega /mc^{2}$. $N=\varepsilon_{0}\frac{VE_{0}^{2}}{2\hbar \omega }$ is the number of photons ℏω in the volume V (where $E_{0}$ and $A_{0}$ are the amplitudes of the electric field and of the vector potential of the e-m radiation, respectively, and $A_{0}=E_{0}/\omega$). The density of the e-m field energy is $\varepsilon_{0}E_{0}^{2}/2$, which translates into number *N* of photons ($\varepsilon_{0}$ is the dielectric constant). As usual for the forced emission, the non-zero value of the probability *w* for N=0 describes the spontaneous emission rate (because the only non-zero matrix element of the creation operator for photons in the case of the quantized vector potential in Equation (12) is out of the diagonal and equal to $\sqrt{N+1}$).

Knowing the probability per time unit of the forced emission *w*, the total number of emitted photons in infinitesimal time duration dt can be written as follows:

(16) dN=(N+1)γdt,

which gives the solution for N(t) in the form lnN=γt. Hence, $lnN_{0}=\gamma \Delta t$, where Δt is the time of the total Fermi sphere decay, and $N_{0}$ is the number of emitted photons being of the order of the particle number in the system. $\Delta t=\frac{lnN_{0}}{\gamma (y)}$ depends on photon frequency $\hbar \omega =ymc^{2}$ via function γ(ℏω) according to Equation (15).

For an exemplary neutron star merger with 2.3 solar masses, the time span Δt of the decay of the Fermi sphere of electrons displays the rapid process of photon emission varying with the ℏω energy of emitted photons as shown in Table 2. However, we do not take into account in the above assessment the time of the decay of neutrons into electrons and protons (and antineutrino), which slows down the photon burst. Such a decomposition ($\beta^{-}$ decay) of neutrons in the initial neutron star is blocked by the Pauli exclusion principle. The $\beta^{-}$ decay of an isolated neutron has the half-time of ca. 880 s and occurs due to theconversion of the negatively charged $-\frac{1}{3}e$ down quark to the positively charged $\frac{2}{3}e$ up quark with the emission of a W^−^ boson. This W^−^ boson decays next, almost instantly, into an electron and an electron antineutrino. If the Pauli exclusion principle is locally waived off, then neutrons decay in a collective manner along the Fermi golden rule, similar to Equation (16) dynamics, with probability $\gamma^{\prime }$ for the spontaneous decay of a neutron. As $\gamma^{\prime }∼\frac{1}{880}$ 1/s, the decay of all neutrons in the considered merger would take ca. 30 h. However, quarks also lose their fermionic quantum statistics when passing the photon sphere rim of a black hole, and the decay of an isolated neutron is here faster—its spontaneous decay into a proton by the conversion d→u of quarks is not hampered by the presence of a second *u* quark in a proton. This accelerates the spontaneous decay of a free neutron by a few orders—to the order of $\gamma^{\prime }∼10^{3}$ 1/s, which leads to the liberation of electrons and protons during the forced collective decay of neutrons in the whole merger of mass 2.3 solar masses within ca. $\ln\left(2.53\times 10^{57}\right)/\gamma^{\prime }≃0.1$ s. The subsequent release of antineutrinos (with the known time scale of $10^{-27}$ s for the decay of bosons W^−^) and of photons (cf. Table 2) are both almost instant. Such timing of the entire process of the merger collapse into a black hole agrees with observations of some kind of short gamma-ray bursts earlier suspected to be associated with the neutron star merger to black hole transitions.

Protons give a slightly longer duration of the burst in the range of high photon energy (cf. Table 2) but also in the ultra-fast time scale. Note also that the total energy of the Fermi sphere of protons in the considered merger is ca. ∼0.7 of the Fermi sphere energy of electrons (at the same Fermi momentum of both, cf. Section 4.2).

The rapid emission of photons at the decay of the Fermi sphere of neutrons during the collapse of unstable neutron star merger is slowed down by the collective $\beta^{-}$-type liberation of the charged (interacting with e-m radiation) components of the initial neutrons. Hence, the photon burst should be associated by a preceding burst of electron antineutrinos emitted at the collective $\beta^{-}$ decay of neutrons. Such antineutrino bursts have not been observed as of yet, which could be linked to the most frequent intergalactic distances to collapsing neutron star mergers and to the almost infinitesimal interaction of antineutrinos with their counters.

### 3.3. The Radiation Efficiency of Fermi Sphere Decay in Plasma at Varying Particle Concentrations

As indicated by Equation (9), the Fermi momentum of fermions in a system depends solely on their concentration $\rho =\frac{N}{V}$. In electrically balanced plasma, where the concentrations of electrons and protons are equal, both types of particles will have the same Fermi momentum.

The energy released per second due to the decay of Fermi spheres of electrons and protons in such plasma can be calculated using Equation (10):

$$E=\left(V/\left(2\pi^{2}\hbar^{3}\right)\right)\int_{0}^{p_{F}}dpp^{2}\left(\sqrt{p^{2}c^{2}+m_{e}^{2}c^{4}}-m_{e}c^{2}+\sqrt{p^{2}c^{2}+m_{p}^{2}c^{4}}-m_{p}c^{2}\right)\mathrm{with}V=N/\rho .$$

Considering that the mass of plasma flowing through the photon sphere rim per second is approximately $M=Nm_{p}++Nm_{e}≃Nm_{p}$, and the energy accumulated in Fermi spheres is equivalent to a mass $E/c^{2}$, we can determine the rate of mass-to-radiation energy conversion:

(17) $$\begin{array}{c} \eta =\frac{E/c^{2}}{M+E/c^{2}}\\ =\frac{NI/(\rho c^{2}2\pi^{2}\hbar^{3})}{Nm_{p}+NI/\left(\rho c^{2}2\pi^{2}\hbar^{3}\right)}\\ =\frac{I}{\rho c^{2}2\pi^{2}\hbar^{3}m_{p}+I}, \end{array}$$

where

(18) $$\begin{array}{c} I=\int_{0}^{p_{F}}dpp^{2}(\sqrt{p^{2}c^{2}+m_{e}^{2}c^{4}}\\ -m_{e}c^{2}+\sqrt{p^{2}c^{2}+m_{p}^{2}c^{4}}-m_{p}c^{2}). \end{array}$$

It is remarkable that η is a function of ρ, solely (via $p_{F}$, the upper limit at integration). Additionally, it is notable that the mass of plasma at the rim of the photon sphere is larger than when far from this region because of the mass increase due to the energy accumulated in the electron and proton Fermi spheres. The accumulation of the fermion kinetic energy in Fermi spheres is undergone at the cost of the attraction of the central black hole in the region outside of the photon sphere, where the Pauli exclusion principle holds and fermions must fill different quantum states in a large ladder of single-particle eigen states. The dependence of the accumulated energy in Fermi spheres on electrons and protons versus particle concentration is illustrated in Table 3. The energy released per second, $E=\frac{V}{2\pi \hbar^{3}}I$, depends on ρ and is proportional to the mass consumption rate per second $M≃\rho Vm_{p}$ (plus also the much lower mass of electrons). This energy released in the form of e-m radiation at the decay of Fermi spheres of charged particles (along the Fermi golden rule, when the Pauli exclusion principle is locally waived out) increases the luminosity of a quasar (or other light sources powered by black holes). Via extracting the non-thermal radiation component of the observed total luminosity (mostly in the X-ray region, which is rather not of the thermal type for black-body temperatures, assessed also from the observations) it is possible to estimate in an independent way the rate of mass consumption, taking into account also the upper limit of the energy of the registered photons (with the help of the data as listed in Table 3 for a few examples). This is similar, to some extent, to the remote measurement of a black-body temperature of an accretion disc via observing an optical thermal part of the radiation and comparing it to a black-body spectrum.

## 4. Discussion

### 4.1. Upgrade of Premises for Information Paradox

The thermodynamics of black holes [1,2] poses the problem of the fate of the information encoded in matter falling into a black hole. Black hole radiation of the Hawking–Unruh type [4,5] is fully random and cannot carry out the information specific to the matter consumed by the black hole, which causes an information paradox. To cope with this paradox, the creation of particle–antiparticle pairs on the event horizon and the escape of a particle associated with the capturing by a black hole of its antimatter partner is considered, which, however, does not solve the problem because of the quantum entanglement property called its monogamy. Particles and antiparticles are entangled, and Hawking radiation must be also entangled in distinct time events [6], and to avoid such a double entanglement, it has been suggested an instant breaking of the particle–antiparticle entanglement at the event horizon of a black hole. Immediately, broken entanglement between the infalling particle and the outgoing antiparticle would release a large amount of energy, creating in this way a hot firewall at the event horizon [6]. Such a hypothetical firewall on the event horizon would not be observable for any distant observer because the falling of the matter on the event horizon takes, for remote observers, an infinite amount of time. Some traces of a fuzzy horizon with a firewall would be noticeable, in principle, via gravitational wave observations. The observational evidence of a fuzzy black hole horizon in a gravitational wave signature gathered by LIGO observations of bouncing black holes [7] is, however, excluded because they are not sufficiently statistically significant [8].

The information paradox poses a problem for quantum gravitation, as Polchinski with co-authors [6] stated that the paradox may eventually require to give up one of three time-tested principles: Einstein’s equivalence principle, unitarity, or the existing quantum field theory. According to the theoretical quantum field models of gravitation singularity, including holographic formulation [3], the unitarity at matter consumption by black holes should be maintained, which challenges, however, Einstein’s equivalence principle or the existing quantum field theory [6].

The decay of quantum statistics when passing the photon sphere rim, presented in this paper, can, however, contribute to the problem of an information paradox and to the concept of a black hole firewall, updating the related premises to some extent. The decay of quantum statics is associated with the release of energy along the Fermi golden rule scheme in agreement with unitary evolution in quantum mechanics. This energy release resembles some kind of firewall positioned, however, at the photon sphere rim instead of a hypothetical one on the event horizon [6]—cf. the scheme in Figure 4. In addition, the event horizon (even if attributed to a firewall) is unobservable for distant observers, as the falling of the matter onto the event horizon takes an infinite amount of time in the Schwarzschild metric, as opposed to the radiation bursts at the photon sphere rim. The latter can be treated as a modified firewall concept. In addition, the decay of Fermi spheres of electrons and protons in plasma is almost instant (cf. Table 2), and slowed down by the $\beta^{-}$ decay of neutrons only upon neutron star collapse. The giant gamma ray bursts associated with the neutron star merger collapses can be thus treated as the observable visualization of a new concept of a black hole firewall located at its photon sphere rim, similarly to the X-ray component of the luminosity of quasars. Although the entropy behavior when passing the photon sphere rim needs a more thorough analysis, the energy escape along with entropy to outer space from the photon sphere rim during quantum statistics transition significantly modifies the premises for the discussion of the information paradox. Moreover, the decay of quantum statistics beneath the photon sphere rim should affect the local quantum field theory formulation in the vicinity of the event horizon inside the photo sphere. The related quantum fields cannot be assigned locally neither as fermionic nor as bosonic, and it raises the question of to what extent this would be helpful in quantum gravity theory formulation.

### 4.2. Astrophysical Observations Which Support Quantum Statistics Transition at Photon Sphere Rim of Black Holes

Superluminous quasars, powered by black holes with masses of approximately one billion solar masses, consume roughly 10 solar masses annually (equivalent to 0.1 Earth mass per second). To explain their exceptionally high luminosity of around $10^{40}$ W over extended periods, these quasars must convert approximately 30% of their accreted mass into electromagnetic radiation. Traditional hydrodynamic models of quasar accretion discs fall short in fully describing such intense radiation, particularly its high-energy photon component [20,21,39,50,51]. A common approach extends the radiation model of the microquasar Cygnus X-1 [19], proposing a hot toroidal region within the accretion disc located at a distance of approximately 6 Schwarzschild radii from the central singularity. In this region, the Comptonization of soft photons could potentially produce sufficiently energetic photons. However, the required plasma temperatures within this bulb—$10^{9}$ K for electrons and $10^{11}$ K for ions—are highly unlikely in the vast accretion discs of superluminous quasars, which have radii approximately $10^{8}$ times larger than that of Cignus X-1. Therefore, the thermal radiation from the accretion disc [52] and not extreme Comptonization cannot explain the radiation intensity and its spectral composition for superluminous quasars [18,20,21,51].

Nevertheless, the decay of Fermi spheres of electrons and protons in quasar accretion discs when passing the photon sphere rim provides an efficient mechanism for converting gravitational energy into electromagnetic radiation, including high-energy photons, which can explain the observed luminosities of giant quasars, avoiding the need for extreme Comptonization.

Matter accreting onto a black hole undergoes high compression and can reach even its maximum stable density at the photon sphere rim in extreme situations. The compression of the infalling matter leads to the accumulation of giant energy within the Fermi spheres of electrons and protons, which in such conditions are quantumly degenerated systems and, before passing the photon sphere rim, constrained by the Pauli exclusion principle. Using Equations (9) and (10), one can estimate the energy accumulated in these Fermi spheres per second in plasma flow. For a typical accretion rate of 0.1 Earth mass per second, the energy released at the photon sphere rim and associated with the decay of Fermi spheres of electrons and protons is approximately $10^{40}$ J per second, primarily in the form of hard X-ray radiation. This non-thermal radiation from the vicinity of the event horizon aligns with the observed luminosities of superluminous quasars, eliminating the need for the Comptonization of soft thermal photons.

General relativistic corrections to the Fermi momentum due to the spacetime curvature near the event horizon are relatively small. These corrections do not significantly alter our energy estimates, as demonstrated by calculations using Equation (10). In the case of extreme stable plasma concentrations near the photon sphere rim, as is believed to occur in superluminous quasars, the efficiency of mass-to-radiation conversion is approximately 30%. This implies that the release of the combined energy of the electron and proton Fermi spheres is ca. $10^{40}$ J per second, equivalent to 30% of the accreted mass (note that this converted into the e-m radiation part of mass multiplied by $c^{2}$ is just the energy accumulated in Fermi spheres in its flow per second due to the plasma compression beyond the photon sphere rim by the gravitational attraction of the singularity).

Electrons have a lower rest mass than protons, leading to a slightly higher total energy in the electron Fermi sphere (both electrons and protons have the same Fermi momentum, as the concentration of both is the same in a neutral plasma). For the same Fermi momentum at the photon sphere rim, the Fermi energy of electrons is approximately 1.4 times that of protons. In both cases, the Fermi energy significantly exceeds the actual temperature of the accretion disc, indicating that the electron and proton Fermi liquids are quantum degenerate near the photon sphere rim.

The Fermi energy of electrons also sets an upper limit on the energy of emitted photons due to Fermi sphere decay. When the matter accretion rate is limited by external factors (like a shortage in matter supply), the plasma density near the photon sphere rim is lower than would be extreme, resulting in reduced energy accumulation in the Fermi spheres and a lower efficiency of mass-to-radiation conversion (also a lower upper limit of emitted photon energy). However, even in these cases, the decay of Fermi spheres still produces non-thermal photons, which can contribute to the radiation from less luminous e-m radiation sources powered by black holes, such as transient active galactic nuclei, microquasars, and tidal disruption events. By fitting the Fermi energy to the observed photon maximum energies in these events, one can estimate the local density of fermions at the photon sphere rim. This information can then be used to calculate the volume of compressed plasma and the matter consumption rate by virtue of the observed total luminosity, providing an independent assessment of the total mass consumed by the black hole during the whole radiation episode. This approach offers a new tool for studying black hole accretion processes and understanding the nature of various astronomical transients. We meticulously analyze the following astronomical events, employing the aforementioned methodology:

- AT 2020neh: A rapidly brightening tidal disruption event (TDE) candidate, originating from a dwarf galaxy SDSSJ152120.07+140410.5 at a redshift of z=0.06. The central black hole within this galaxy possesses a mass of approximately $10^{5}$ solar masses [54]. AT 2020neh has been under continuous observation since June 2020, with its peak luminosity occurring in July and persisting for approximately 20 days. Concurrent observations in optical, ultraviolet, and X-ray wavelengths have revealed an X-ray component reaching a maximum of $4.5\times 10^{34}$ W, comparable to other TDEs [55]. The overall peak luminosity attained $4.2\times 10^{36}$ W. By employing conventional hydrodynamic models [56], it has been estimated the rate at which stellar debris from the disrupted star (assumed to be a main sequence star with a mass of 1.3 solar masses) is accreting onto the black hole. The observed optical and ultraviolet spectra have been successfully fitted using a black body temperature model, exhibiting a cooling rate of approximately $10^{4}$ K per 20 days. While no definitive mechanism for the X-ray component has been identified, post-flare observations and prior survey data have ruled out the presence of a gaseous accretion disk around the host galaxy’s black hole. However, by applying Equation (10) to the disrupted stellar debris during the peak luminosity phase and considering the maximum energy of observed X-ray photons (10 keV), we find that the contribution to the luminosity primarily in the X-ray range is approximately $4\times 10^{34}$ W due to the decay of Fermi spheres within the ionized debris consumed by the black hole. This result is consistent with the observed spectra of TDEs [54,55].
- AT2021lwx: Located at a redshift of z=0.995, AT2021lwx exhibited a temporal increase in radiation luminosity to $7\times 10^{38}$ W, commencing in April 2021 and lasting over a year [57]. This event represents the most energetic non-quasar transient ever observed, with no prior emission detected in the preceding several years. The optical and ultraviolet spectral energy distribution of AT2021lwx indicates a black-body temperature of $1.2\times 10^{4}$ K. This transient is believed to be the result of a massive gaseous cloud accreting onto a black hole (this black hole mass has been estimated to be between $10^{8}$ and $10^{9}$ solar masses), rather than TDE. The observed X-ray component, spanning the energy range of 0.3 to 10 keV with a luminosity of $1.52\times 10^{38}$ W, has proven challenging to explain using conventional models at such a low temperature. However, by incorporating the emission arising from the decay of Fermi spheres within compressed fermion systems passing through the photon sphere rim of the central black hole, we can reconcile the Fermi momentum (related to the compression level via Equation (9) and linked to the maximum observed photon energy) and a realistic supply of matter at the accretion site to achieve a luminosity of approximately $1.5\times 10^{38}$ W. This contribution effectively supplements the total luminosity of AT2021lwx and provides an explanation for the observed X-ray component at this event.
- Transient AGN 1ES 1927+654: Located at a redshift of z=0.017, this active galactic nucleus (AGN) underwent a 100-fold brightening event lasting for one year, beginning in December 2017. The optical and ultraviolet spectral components increased during this period, while the gamma-ray flux exhibited a decline. By July 2018, the X-ray coronal emission had vanished, only to reappear a few months later [58]. The source subsequently returned to its pre-changing-look state after approximately one year in the optical, ultraviolet, and X-ray ranges. Previous interpretations [58] suggested that the puzzling behavior of gamma-ray radiation in AGN 1ES 1927+654 was due to the temporary quenching of jets from a spinning black hole caused by the consumption of an oppositely magnetized gas cloud during a changing-look episode. However, the quantum statistics transition at photon sphere rim of the black hole offers an alternative explanation for the observed behavior without relying on such speculations about jet quenching. In the case of AGN 1ES 1927+654, the source of gamma radiation is believed to be associated with electrons and positrons accelerated by the magnetic field within the jets of the spinning black hole, in accordance with the Blandford–Znajek model of jet formation [59]. This model explains the formation of jets for spinning Kerr-like black holes, where the dragging of the reference frame in the Kerr metric causes the magnetic field frozen within the accretion matter to rotate. This results in an outgoing flux of angular momentum, extracting energy from the system and propelling jets into outer space. The rotating magnetic field within these jets accelerates electrons and positrons, leading to the production of gamma-ray radiation. The source of electron–positron pairs in the Blandford–Znajek model is a strong electric field generated by the rotating magnetic field frozen within the ergosphere. In the case of the AGN 1ES 1927+654 transient, the decay of Fermi spheres of electrons and protons from the occasionally captured gas cloud produces sub-MeV radiation (at most 2 keV, consistent with X-ray observations [58]) due to the relatively low plasma compression at the photon sphere rim. These photons are unable to excite additional electron–positron pairs within the ergosphere but can exert a force on existing pairs created according to the Blandford–Znajek mechanism, pushing them towards the event horizon. This ultimately leads to a reduction in the supply of electrons and positrons to the jets (via diffusion to jets across nodes in the ergosphere of a Kerr-like black hole), resulting in a temporary quenching of gamma-ray radiation without the need to speculate on the demagnetization of the AGN by an oppositely magnetized gas cloud during this episode [58].

The aforementioned examples provide compelling support for the model of Fermi sphere decay in matter compressed by the central black hole as it traverses the photon sphere rim. Moreover, the described quantum transition is predicted to terminate rapidly upon crossing this boundary, in accordance with the Fermi golden rule as outlined in Section 3.2. This rapid termination could potentially refine conventional models of radiation decline observed in AGN transients and tidal disruption events.

## 5. Conclusions

The local departure from the Pauli exclusion principle in multi-particle systems approaching the event horizon of a black hole when passing the photon sphere rim [43,45] explains the release of short giant gamma-ray bursts at collapses of neutron star mergers exceeding the TOV limit. Both the energy and timing of these bursts agree with the energy accumulated in the Fermi sphere of neutrons and with the time rate of its decay. The same quantum mechanism contributes to the luminosity of quasars with additional X-ray non-thermal radiation from the vicinity of the event horizon to well below the inner edge of the accretion disc assumed in conventional models [19,39,50]. The mass-to-radiation energy conversion rate at Fermi sphere collapse reaches 30% for the uppermost electron–hadron concentration in stable compressed matter (like in a neutron star at TOV limit or in superluminous quasars). Such a situation corresponds to the supply of matter to a black hole limited only by the stability of compressed matter, and in cases of lower matter supply, the effect of the Fermi sphere decay is less powerful but still can contribute to the luminosity of micro-quasars and transients in closer active galactic nuclei (AGNs) or even at flares associated with tidal disruption events (TDEs). The decay of the Fermi sphere mainly produces X-rays (even during not extreme matter compression), which can conveniently complement conventional models of AGN transients and TDE bursts, improving the fit to the observational data.

The described topological effect contributes to the problem of the information paradox and the related concept of the firewall of a black hole [1,3,6]. The photon radiation associated with the decay of structured collective multi-particle systems of fermions when passing the photon sphere rim of a black hole offers a mechanism of a firewall, allowing the avoidance of the problem with the unitarity violation upon matter consumption by a black hole.

## Figures and Tables

**Table 1:** For a neutron star merger with M=2.3 $M_{⊙}$ (only the rest mass of neutrons, $N=2.73\times 10^{57}$ of neutrons), a varying radius *r* is assumed. The corresponding neutron concentration $\rho =\frac{N}{V}$ and density $d=\frac{M+E/c^{2}}{V}$ (including the mass increase due to energy accumulated in the neutron Fermi sphere). For such a concentration of neutrons, their Fermi momentum $p_{F}$ is assessed. The energy accumulated in the Fermi sphere in the compressed neutron star merger *E* is equivalent to a fraction of the sun mass $\frac{E}{c^{2}}$ (this energy is of the order of the total energy of the short giant gamma-ray burst associated with a neutron star merger collapse).

Radius *r*	$\rho =\frac{N}{V}$, $N=\frac{M}{m_{n}}$	$d=\frac{M+E/c^{2}}{V}$	$p_{F}$ [kg m/s]	*E* [J]	$\frac{E}{c^{2}}$
6 km	$3.2\times 10^{45}$ 1/m^3^	$5.6\times 10^{18}$ kg/m^3^	$4.7\times 10^{-19}$	$4.75\times 10^{46}$ J	0.26 $M_{⊙}$
8 km	$1.27\times 10^{45}$ 1/m^3^	$2.27\times 10^{18}$ kg/m^3^	$3.5\times 10^{-19}$	$2.7\times 10^{46}$ J	0.15 $M_{⊙}$
10 km	$6.5\times 10^{44}$ 1/m^3^	$1.14\times 10^{18}$ kg/m^3^	$2.8\times 10^{-19}$	$1.84\times 10^{46}$ J	0.1 $M_{⊙}$

**Table 2:** Fermi golden rule estimation of the time Δt of complete decay of the Fermi sphere for neutron star merger with the mass 2.3 M_⊙_ as in Table 1 for different photon energies ℏω and for electron and proton contributions.

	Electrons		Protons	
**ℏω [eV]**	**γ(ℏω) [1/s]**	**Δt [s]**	**γ(ℏω) [1/s]**	**Δt [s]**
0.1 GeV	$2.5\times 10^{22}$	$5\times 10^{-21}$	$2.9\times 10^{21}$	$4.5\times 10^{-20}$
1 MeV	$2.4\times 10^{26}$	$5\times 10^{-25}$	$2.4\times 10^{24}$	$5.5\times 10^{-23}$
1 keV	$2.6\times 10^{28}$	$5\times 10^{-27}$	$2\times 10^{30}$	$6\times 10^{-29}$

**Table 3:** The Fermi energy, denoted by $\varepsilon_{F}$, for electrons in a system determines the maximum energy $\hbar \omega_{max}$ of emitted photons. Associated with this energy is the Fermi momentum $p_{F}$, which represents the radius of the Fermi sphere in momentum space. The relation between the Fermi energy and Fermi momentum is given by $\varepsilon_{F}=\sqrt{p_{F}^{2}c^{2}+m_{e}^{2}c^{4}}-m_{e}c^{2}$. The Fermi momentum, in turn, is directly related to the electron concentration ρ as $p_{F}=\hbar {\left(3\pi^{2}\rho \right)}^{1/3}$; cf. Equation (9). In a neutral plasma passing through a photon sphere rim, the rate of mass-energy conversion η during the decay of Fermi spheres of electrons and protons depends solely on the particle concentration ρ at that rim (the same for electrons and protons); cf. Equation (17). The luminosity due to this process is calculated for a specific Fermi energy and a mass consumption rate by a black hole—an exemplary one of 0.06 Earth masses per second (or 5.6 solar masses per year). The luminosity is proportional to this mass consumption rate.

$\varepsilon_{F}$ [eV]	$p_{F}$ [kg m/s]	$\rho =\frac{N}{V}$ [1/m^3^]	η [%]	Luminosity
2 keV	$2.4\times 10^{-23}$	$4\times 10^{32}$	0.00006	$2\times 10^{34}$ W
10 keV	$5.4\times 10^{-23}$	$4.6\times 10^{33}$	0.0003	$1\times 10^{35}$ W
100 keV	$1.7\times 10^{-22}$	$1.4\times 10^{35}$	0.003	$1\times 10^{36}$ W
1 MeV	$7.5\times 10^{-22}$	$1.2\times 10^{37}$	0.04	$1.2\times 10^{37}$ W
10 MeV	$5.6\times 10^{-21}$	$5.1\times 10^{39}$	0.4	$1.3\times 10^{38}$ W
100 MeV	$5.3\times 10^{-20}$	$4.3\times 10^{42}$	4	$1.3\times 10^{39}$ W
0.5 GeV	$2.7\times 10^{-19}$	$5.7\times 10^{44}$	20	$5.9\times 10^{39}$ W
0.8 GeV	$4.2\times 10^{-19}$	$2.3\times 10^{45}$	29	$1.2\times 10^{40}$ W
1 GeV	$5.3\times 10^{-19}$	$4.3\times 10^{45}$	35	$1.7\times 10^{40}$ W

## Data Availability

The original contributions presented in the study are included in the article; further inquiries can be directed to the corresponding author.

## Appendix A. Quantum Statistics and Trajectories Entering Feynman Path Integrals

Quantum statistics of indistinguishable identical particles is rooted in topology [25,27,32,40] and is defined by a scalar unitary representation of the braid group $\pi_{1}\left(F_{N}\right)$ for a particular system of *N* particles with dynamics confined to some spatial manifold M. For these particles, the multi-particle configuration space is defined by Equation (7), i.e., as $F_{N}=\left(M^{N}-\Delta \right)/S_{n}$. In this definition, $M^{N}=M\times M\times \cdots \times M$ is an *N*-fold product of the manifold to account, independently, for the positions of all particles. Δ denotes a diagonal subset of $M^{N}$ collecting points, for which coordinates of at least two particles coincide, and subtracted from the space in order to ensure particle number conservation. The quotient structure of the space $F_{N}$ is generated via the division by permutation group $S_{N}$, which displays the indistinguishability of identical particles. The division by $S_{n}$ means that particle distributions on M, which differ by particle numeration only, are unified into a single point in $F_{N}$. The topological structure of the space $F_{N}$ is characterized by homotopy groups [23], in trajectory terms, by the first homotopy group $\pi_{1}$. This group for an arc-connected space A, $\pi_{1}\left(A\right)$, collects non-homotopic classes of closed trajectory loops in A. Non-homotopy [23] means that trajectory loops from different classes cannot be deformed in a continuous manner one onto another without cutting. If $\pi_{1}\left(A\right)=\left\{e\right\}$, where *e* is a neutral group element, then the space A is simply connected. Otherwise, when $\pi_{1}\left(A\right)$ is non-trivial, the space A is multiply connected. In the case where for the space A the multi-particle configuration space $F_{N}$ is taken, the first homotopy group $\pi_{1}\left(F_{N}\right)$ is called the braid group [25,26], and trajectory loops are multi-strand paths in $F_{N}$ joining variously numbered but the same distribution of particles. These braids display exchanges of particles on M. Braid entanglements of individual particle trajectories in multi-strand bunches depend on the topology of M, including the dimension of M. For 3D spatial manifolds in unfolded spacetime, $\pi_{1}\left(F_{N}\right)=S_{N}$, the finite permutation group of $\mathrm{Dim}S_{N}=N!$. For 2D spatial manifolds, $\pi_{1}\left(F_{N}\right)$ is different. For $M=R^{2}$, $\pi_{1}\left(F_{N}\right)=B_{N}$, where $B_{N}$ is an infinite (countable) Artin group [26,30].

Quantum statistics is assigned to indistinguishable particles according to scalar unitary representations of the braid group for a particular system. Various braid groups have different scalar unitary representations, and these representations are usually multiple. The permutation group $S_{N}$ has two different scalar unitary representations, defined by group generators $\sigma_{i}\to e^{i\pi }$ and $\sigma_{i}\to e^{i0}$, where $\sigma_{i}$, i=1,⋯,N−1 are elementary braids (group generators) describing exchanges of the *i*-th particle with the (i+1)-th one when other particles remain at rest. These two representations define fermions and bosons, respectively. The Artin group $B_{N}$ has an infinite number of different scalar unitary representations, $\sigma_{i}\to e^{i\alpha }$, α∈[0,2π), which define fractional quantum statistics and related quantum particles called anyons, besides fermions and bosons [31].

The definition of quantum statistics via a choice of a scalar unitary representation of a particular braid group is formulated mathematically within the quantization via Feynman path integration. The latter was defined originally for a single particle [34] and subsequently generalized for multi-particle systems of indistinguishable particles [27,28,29,32]. The Feynman integral over trajectories for *N* particles on a manifold M [27,34,41,42] gives the matrix element of the quantum evolution operator of a whole system in position representation,

(A1) $$\begin{array}{c} I(Z_{1},t_{1};Z_{2},t_{2})\\ =\sum_{l}e^{i\alpha_{l}}\int d\lambda_{l}e^{iS[\lambda_{l}\left(Z_{1},t_{1};Z_{2},t_{2}\right)]/\hbar }, \end{array}$$

where points $Z_{1}=\left(z_{1}^{1},\ldots ,z_{N}^{1}\right)$ and $Z_{2}=\left(z_{1}^{2},\ldots ,z_{N}^{2}\right)$ are two different points in multi-particle configuration space $F_{N}$ of *N* indistinguishable particles, which define the start and final points for the propagator (matrix element of the quantum evolution operator), $I(Z_{1},t_{1};Z_{2},t_{2})$, between localized states $Z_{1}$ at time instant $t_{1}$ and $Z_{2}$ at $t_{2}$, respectively. The summation over trajectories (integration with the measure $d\lambda_{l}$ in the space of trajectories) concerns all accessible classical trajectories $\lambda_{l}$ linking points $Z_{1}$ and $Z_{2}$ in $F_{N}$. Both the trajectory and the measure are assigned with an index *l*, numbering braids from the braid group. This denotes an attachment of the *l*-th braid loop to an open trajectory joining $Z_{1}$ and $Z_{2}$ in $F_{N}$, and displays the possibility of the exchanges of indistinguishable particles in the way of a multi-strand trajectory. According to the group structure, an arbitrary number of braids added to an open trajectory in arbitrary intermediate points of this open trajectory add up to a single braid indexed by *l*—a discrete index because braid groups are countable as multi-cyclic groups generated by finite set of generators $\sigma_{i}$. The classical action functional $S[\lambda_{l}\left(Z_{1},t_{1};Z_{2},t_{2}\right)]$ is the time integral of Lagrangian $\int_{t_{1}}^{t_{2}}L\left[\lambda_{l}\right]dt$, where L=T−V, and *T* is the kinetic energy of all particles and *V* is their potential energy including interparticle interaction. $S[\lambda_{l}]$ is a functional over the domain of trajectories. This domain is decomposed into disjoint sectors numbered by *l* because of the non-homotopy of braids. The measure for the integration over trajectories thus splits into a family of independent measures $\left\{d\lambda_{l}\right\}$ because the measure definition requires a continuity condition on the domain, which is impossible to be satisfied for trajectories with added different braids that are mutually non-homotopic. In this way, the Feynman path integral splits into integration over disjoint path domain sectors and the contributions of all sectors must be added up with arbitrary weight factors (unitary due to the causality requirement for quantization). These weight factors form a scalar unitary representation of the braid group [27] denoted in Equation (A1) as $e^{i\alpha_{l}},\;\mathrm{with}\;\alpha_{l}\in \left[0,2\pi \right)$. The choice among various possible braid group representations determines the quantum statistics of particles after quantization.

The sum over trajectories in the Feynman path integral is precisely defined by the domain of the measure in the path space. The measure $d\lambda_{l}$ (for each *l*) is constructed in analogy to the Wiener measure [44] for the path integration of stochastic processes, used earlier to describe Brownian motion [41,44]. Feynman path integrals differ from Wiener integrals in complex valued integrands [34,41]. Nonetheless, the requirements imposed onto trajectories included in the measure domain are similar to those for the Wiener measure, i.e., trajectories must be classically accessible piece-wise continuous paths for particles [34]. Any trajectory contributing to the path integral arises via the construction of the measure in the Feynman path integral (cf. Chapter 2.4 in Ref. [34]) related to the discretization of time and the building of various piece-wise continuous curves from pieces of arbitrary classical trajectories on infinitesimal time segments. This procedure is conditioned only by the existence of real classical trajectories on particular segments, though the resulting continuous non-smooth trajectory is not, in general, an admissible classical trajectory. Integrating over arbitrary positions of intermediate discretization points (for the infinitesimal step of the time discretization) means the sum over arbitrary trajectories. In the case when some real classical trajectories are inaccessible in a system, the construction of the measure is ineffective, and related trajectories must be discarded from the path domain. This has been discussed for Brownian motion with barriers and noticed for Feynman path quantization by Pauli [41].

The measure construction concerns also braid paths in the case of the multi-particle system of indistinguishable particles. If braids do not exist due to a topological reason, like being beneath the photon sphere rim of a black hole, then the sum over *l* disappears from Equation (A1), and the factor $e^{i\alpha_{l}}=1$, which does not assign any quantum statistics (if the braid group is trivial, $\pi_{1}\left(F_{N}\right)=\left\{e\right\}$, its scalar unitary representation is only e→1, as e·e=e, and this representation does not assign any statistics because *e* does not describe any interchange of particles).

## Appendix B. Quantum Statistics and Spin: A Topological Perspective

The Pauli theorem [60], one of the cornerstones of quantum mechanics, posits a direct correlation between spin of particles and their quantum statistics. Half-integer spin particles are fermions, while integer spin particles are bosons. This theorem’s foundation lies in the Dirac electrodynamics for $\frac{1}{2}$-spin non-interacting particles, where the Hamiltonian formulation necessitates anticommuting quantum fields for positive-definite kinetic energy [61,62]. Pauli’s theorem extends to interacting particles and can be elegantly proven through a topological framework using homotopy group for quantum statistics definition [25,40]. This connection arises from the overlap between the rotation group, defining spin or angular momentum, and the braid group, describing quantum statistics, resulting in the coincidence of their unitary representations.

In 3D manifolds, the rotation group O(3) is covered by SU(2), yielding two classes of irreducible unitary representations corresponding to integer and half-integer angular momenta [63]. These align with the two possible scalar unitary representations of the permutation group $S_{N}$, which is the braid group for 3D manifolds. The overlap between these groups ensures their representation compatibility. Consequently, half-spin representations of the rotation group must correspond to the odd representation $\sigma_{i}\to e^{i\pi }=-1$ of the braid group (fermions), while integer angular momentum representations align with the even representation $\sigma_{i}\to e^{i0}=1$ of the permutation group (bosons).

This topological approach extends Pauli’s theorem to 2D manifolds, encompassing anyons, particles that defy the fermion–boson dichotomy [29,32]. In 2D, the Abelian rotation group O(2) is isomorphic to U(1), sharing the same continuous scalar unitary representations $e^{i\alpha }$, α∈[0,2pi) as the Artin group (the braid group for $M=R^{2}$). Therefore, Pauli’s theorem holds even for non-quantized spin $s=\frac{\alpha }{2\pi }$ in 2D (where rotations commute), aligning with anyon statistics defined by $e^{i\alpha }$ (α∈[0,2π)).

The equivalence of quantum statistics and spin, however, hinges on the simultaneous existence of a rotation group and a braid group that are both non-trivial. When one of these groups is trivial, as in the case of a simply connected configuration space $F_{N}$ beneath a black hole’s photon sphere rim, the spin may be defined independently of the statistics. This occurs due to the vanishing overlap between the trivial braid group and the rotation group (except for a neutral element), circumventing the Pauli theorem’s restrictions.
